# Arachidonic Acid Cascade and Eicosanoid Production Are Elevated While LTC4 Synthase Modulates the Lipidomics Profile in the Brain of the HIVgp120-Transgenic Mouse Model of NeuroHIV

**DOI:** 10.3390/cells11132123

**Published:** 2022-07-05

**Authors:** Nina Y. Yuan, Ricky Maung, Ziying Xu, Xianlin Han, Marcus Kaul

**Affiliations:** 1 Division of Biomedical Sciences, School of Medicine, University of California Riverside, 900 University Ave, Riverside, CA 92521, USA; nina.yuan@medsch.ucr.edu (N.Y.Y.); ricky.maung@medsch.ucr.edu (R.M.); 2 Infectious and Inflammatory Disease Center, Sanford Burnham Prebys Medical Discovery Institute, 10901 North Torrey Pines Road, La Jolla, CA 92037, USA; 3 Barshop Institute for Longevity and Aging Studies, University of Texas Health San Antonio, San Antonio, TX 78229, USA; xuz1@uthscsa.edu (Z.X.); hanx@uthscsa.edu (X.H.); 4 Department of Medicine-Diabetes, University of Texas Health San Antonio, San Antonio, TX 78229, USA

**Keywords:** HIV, HANDs, arachidonic acid, eicosanoids, LTC4S, lipidomics, neuroinflammation, sexual dimorphism, mRNA, cell signaling

## Abstract

Background: Combination antiretroviral therapy (cART) has transformed HIV infection from a terminal disease to a manageable chronic health condition, extending patients’ life expectancy to that of the general population. However, the incidence of HIV-associated neurocognitive disorders (HANDs) has persisted despite virological suppression. Patients with HIV display persistent signs of immune activation and inflammation despite cART. The arachidonic acid (AA) cascade is an important immune response system responsible for both pro- and anti-inflammatory processes. Methods: Lipidomics, mRNA and Western blotting analysis provide valuable insights into the molecular mechanisms surrounding arachidonic acid metabolism and the resulting inflammation caused by perturbations thereof. Results: Here, we report the presence of inflammatory eicosanoids in the brains of a transgenic mouse model of NeuroHIV that expresses soluble HIV-1 envelope glycoprotein in glial cells (HIVgp120tg mice). Additionally, we report that the effect of LTC4S knockout in HIVgp120tg mice resulted in the sexually dimorphic transcription of COX- and 5-LOX-related genes. Furthermore, the absence of LTC4S suppressed ERK1/2 and p38 MAPK signaling activity in female mice only. The mass spectrometry-based lipidomic profiling of these mice reveals beneficial alterations to lipids in the brain. Conclusion: Targeting the AA cascade may hold potential in the treatment of neuroinflammation observed in NeuroHIV and HANDs.

## 1. Introduction

After 40 years since its emergence in the United States, human immunodeficiency virus (HIV) continues to challenge the medical and scientific community. As of 2020, the UNAIDS has estimated that globally the number of people infected with HIV since the start of the epidemic has reached 79.3 million people—with those currently living with HIV accounting for 37.7 million individuals [1]. The proportion of people living with HIV and older than 45 years of age is more than 60% in the United States [2], in large part due to the advent of antiretroviral therapy. The development of effective combined antiretroviral therapy (cART) has had enormous benefits in survival and increased life expectancy of those living with HIV. However, the neurocognitive manifestations of HIV infection, also known as HIV-associated neurocognitive disorders (HANDs), have continued to occur in 30–50% of infected people, despite effective virological suppression with cART [3,4,5]. Several longitudinal studies have revealed that the majority of patients with HANDs and on cART therapy have stable neurocognitive status that neither improves nor declines [6,7,8,9], suggesting that effective viral suppression or even the eradication of the virus may not necessarily improve the neurocognitive decline brought about by the disease. Given that advanced age is associated with reduced neurocognitive performance and susceptibility to HANDs [10,11,12,13,14,15,16,17] and the challenges of developing either a cure or a functional cure for HIV, it is critical to find solutions to HANDs independent of HIV-viral infection.

Lipids and their metabolism play a crucial role in the function and development of the brain [18,19]. The pathways by which lipids are controlled are tightly regulated and the perturbation of any component or stepwise processing could lead to overarching alterations in lipid homeostasis. Eicosanoids are a group of bioactive oxidized signaling lipids derived from arachidonic acid and other polyunsaturated fatty acids (PUFAs). Classical eicosanoids are well characterized for their role in acute inflammation and high pro-inflammatory mediation in the immune response. However, these oxidized fatty acid derivatives function in diverse roles in both homeostatic regulation as well as the resolution of inflammatory processes [20]. Canonically, the biosynthetic pathway of eicosanoids begins with arachidonic acid (AA) which can be further metabolized via multiple routes; including the cyclooxygenase (COX) pathway to form prostaglandins (PG) and thromboxane (TX), or via the lipoxygenase (LOX) pathway to form leukotrienes (LTs) and hydroxyeicosatetraenoic acids (HETEs) through conversion by 5-lipoxygenase (5-LOX) and other members of the LOX family (8-,12-,15-LOX). Alternatively, the metabolism of AA can also occur through a cytochrome P450 epoxyhydrolase (CYP)-mediated pathway to form HETEs and epoxyeicosatrienoic acids (EETs). This process is cumulatively known as the arachidonic acid cascade. These lipid mediators are not stored in cells in large amounts but synthesized de novo on site. The pro-inflammatory products from both the COX and 5-LOX pathways were implicated in a number of diseases and disorders, making them a popular subject of research for pharmaceutical targeting [21].

Multiple findings have pointed to the importance of eicosanoids in NeuroHIV and HANDs [22,23,24,25,26,27,28,29,30,31,32,33,34,35,36,37]. Previous work by Albright and Gonzalez-Scarano implicated leukotriene C4 synthase (LTC4S) in the HIV infection of human glial cells and our lab found leukotriene C4 synthase (LTC4S), 5-LOX and cysteinyl LT receptor 1 (Cysltr1) to be differentially regulated in the brains of HIV glycoprotein 120 (HIVgp120) expressing transgenic mice (HIVgp120tg), which is an animal model for NeuroHIV [37,38]. LTC4S is a downstream enzyme of 5-LOX and directly leads to the biosynthesis of leukotrienes LTC4, LTD4, and LTE4—collectively termed cysteinyl leukotrienes (cysLTs). Herein, we describe the presence of inflammatory eicosanoids in the brains of HIV-gp120tg mice. Additionally, we report that the effect of LTC4S knockout in HIVgp120tg mice resulted in sexually dimorphic transcription of COX- and 5-LOX-related genes. Furthermore, the absence of LTC4S suppressed ERK1/2 and p38 MAPK signaling activity in female mice only. The mass spectrometry-based lipidomic profiling of these mice reveals the possible benefits of HIV-mediated lipid alterations, suggesting targeting the AA cascade may hold potential in the treatment of the neuroinflammation observed in NeuroHIV and HANDs.

## 2. Materials and Methods

### 2.1. Mice

HIVgp120tg mice were kindly provided by Dr. Lennart Mucke (Gladstone Institute of Neurological Disease, University of California, San Francisco, CA, USA) [39]. These HIVgp120tg mice expressed a soluble viral envelope protein gp120 of the CXCR4-using HIV-1 LAV isolate which infects lymphocytes and macrophages [40]. Two founder lines of HIVgp120tg mice (L1 and L2) were characterized in terms of CNS gene expression and the role of CCR5 in brain injury associated with the expression of the viral envelope protein. The latter L2 line (CCR5WT, hereafter referred to as HIVgp120tg) was selected for this study for its 1.5-fold higher RNA expression of the gp120 transgene at 1.5 months of age, a difference which dissipates by 3 months of age [38]. The HIVgp120tg mice were well characterized and manifest neuropathological features by 6 months of age and neurocognitive impairment by 12 months of age, comparable to that observed in NeuroHIV/AIDS patients. The persistent neuroinflammatory markers and lack of active viral infection makes this model suitable for the study of long-term virally suppressed patients [41]. Mice-deficient in functional LTC4S [42] were kindly provided by Dr. Yoshihide Kanaoka (Brigham and Women’s Hospital, Boston MA). LTC4SKO mice were crossbred with HIVgp120tg mice and the F3 generations of HIVgp120tg^het^-Ltc4s^het^ mice were used to generate four distinctive experimental genotypes: WT (wildtype control, HIVgp120tg^−/−^-Ltc4s^+/+^), HIVgp120tg (HIVgp120tg^+/+^-Ltc4s^+/+^), LTC4SKO (HIVgp120tg^−/−^-Ltc4s^−/−^) and LTC4SKO/HIVgp120tg (HIVgp120tg^+/+^-Ltc4s^−/−^). All mice were in a mixed SJL/C57BL6/B6;129P genetic background. The tissues were collected following terminal anesthesia and cardiac perfusion with 0.9% saline solution. All mice were genotyped by PCR using genomic DNA from tail biopsies as described [38,42]. For primers, see Table 1. Whole brain tissue was assessed for eicosanoid quantification and Western blot to ensure the detection of low abundance analytes. Regiospecific tissue such as the cerebral cortex and hippocampus were analyzed via qPCR and shotgun lipidomics, as histopathological studies of these brain structures have shown marked neurodegeneration and are most prominently affected in HIV patients with neurocognitive impairment [39]. All experimental procedures and protocols involving animals were in accordance with NIH guidelines and approved by the Institutional Animal Care and Use Committee of the Sanford Burnham Prebys Medical Discovery Institute (AUF 15-035, approved 4/17/2015) and the University of California, Riverside (AUP 20170048, approved 12/5/2017, AUP 20200041, approved 11/3/2020).

### 2.2. Quantitative Analysis of Eicosanoids in Mouse Brain Tissue via LCMS

Whole brain tissue was collected from two cohorts consisting of a 6-month-old set made up of *n* = 6 wild type (WT), *n* = 6 HIVgp120tg mice (3 males and 3 females per genotype) as well as a 12-month set consisting of *n* = 6 wildtype, *n* = 6 HIVgp120tg mice (3 males and 3 females per genotype). Samples were snap-frozen in liquid nitrogen and stored at −80 °C until they were shipped and processed and analyzed by Cayman Chemical as a commercially available service [43]. Briefly, frozen brain samples were homogenized in 0.83 mL of PBS using Precellys tissue homogenizer in CK14 bead containing tubes (Precellys) for 2 cycles of 30 s at 5800 rpm with a 30 s pause in between. The homogenate was then spiked with internal standard solution with 0.83 mL of methanol containing 307 pg of the deuterated internal standard controls of leukotriene C4-ds, D4-ds, E4-ds (Cayman 10006198, 10006199). The resulting homogenate was incubated in a −80 °C freezer for a few hours to improve extraction. Samples were centrifuged at 15,000× *g* for 10 min before loading onto a solid-phase extraction 96-well plate (Polymeric reversed phase, 10-mg (Phenomenex, Torrance, CA, USA)), previously activated with 2 mL methanol and rinsed with 2 mL water. The samples as well as a freshly prepared standard curve consisting of Leukotriene C4, D4, E4 (Cayman 20210, 20310, 20410) were diluted upon loading so that the final concentration of methanol was between 10 and 15% of the total volume. After washing with water, extracts were eluted with 1 mL methanol. Solvent was then evaporated under vacuum in a SpeedVac centrifuge and the samples were resuspended in 100 µL water/acetonitrile 60:40 (*v*/*v*). An aliquot of 10 µL, equivalent to the extract of 4.7 µg of brain tissue, was injected into the LC-MS/MS system (Sciex ExionLC Integrated System with Sciex Triple Quad 6500+ (Sciex, Framingham, MA, USA) for analysis. The chromatographic profile of the ion count for each *m*/*z* transition was monitored and the area under the peak (ion intensity vs. elution time) was integrated using commercial software (MultiQuant, Sciex). The mass transitions and collision energies used are listed in Supplementary Table S1. Concentrations were quantified using the area ratios analyte/internal standard in picograms per 10 µL. All measured analytes were within detection limits with the exception of LTE4 which was not detected. Measurements between the 6-month and 12-month cohorts were compared in GraphPad Prism with an unpaired *t*-test and differences among the different age groups of the same genotype were found to be non-significant and thus measurements were pooled between genotypes for a total of *n* = 12 for WT and HIVgp120tg (6 males and 6 females per genotype).

### 2.3. qRT-PCR

Tissue from the cortex of a 12 month-old cohort consisting of *n* = 3 per genotype for both male and female mice were collected for mRNA extraction. Whole-cortex RNA were isolated using the Qiagen RNeasy Lipid Tissue Midi Kit (Qiagen, Germantown, MD, USA; cat# 75144) according to the manufacturer’s instructions. Five hundred nanograms (500 ng) of RNA was then synthesized into cDNA by SuperScript II reverse transcriptase (Thermo Fisher Scientific, Waltham, MA, USA; cat# 18064014) as per the manufacturer’s instructions. cDNA was then treated with RNase H (Thermo Fisher Scientific, cat# 18021071) for 20 min at 37 °C and stored at −20 °C until use. Quantitative real-time polymerase chain reaction (qRT-PCR) was performed using Power PCR SYBR Green Master Mix (Thermo Fisher Scientific, cat# 4368708) and the primers listed in Table 1. QRT-PCR was performed using a QuantStudio 6 Flex system (Applied Biosystems/Life Technologies, Waltham, MA, USA) and ∆∆Ct values were calculated using the housekeeping gene GAPDH as previously described [38,44]. ANOVA analyses of male and female mice were performed independently. For visualization purposes and to illustrate the sex differential expression and detection, data were normalized to the male wildtype expression values of the gene.

### 2.4. Western Blot

Whole brain tissue was collected from a 12-month-old cohort consisting of *n* = 6 animals per genotype for both male and female mice. Tissues were processed as previously published with minor modifications [38,44,45]. Fifty micrograms of protein were loaded on a 4–12% SDS-PAGE gel (Invitrogen, Waltham, MA, USA) and transferred onto PVDF membrane (Invitrogen) with SeeBlue Plus2 pre-stained protein standard ladder (Invitrogen). Antibodies phospho-p38 (1°Ab- 1:1000; anti-rabbit 2°Ab- 1:5000, 43 kDa) (Cell Signaling, Danvers, MA, USA; 9211), total p38 (1°Ab- 1:2000; anti-rabbit 2°Ab- 1:25,000, 40 kDa) (Cell Signaling; 9212), phospho-ERK1 (1°Ab- 1:1000; anti-rabbit 2°Ab- 1:5000, 42/44 kDa) (Cell Signaling; 9101), total ERK1 (1°Ab- 1:2000; anti-rabbit 2°Ab- 1:5000, 42/44 kDa) (Cell Signaling; 9102), active JNK (1°Ab- 1:1000; anti-rabbit 2°Ab- 1:3000, 46/54 kDa) (Promega, Madison, WI, USA; V793A), total JNK (1°Ab- 1:1000; anti-rabbit 2°Ab- 1:3000, 46/54 kDa) (Cell Signaling; 9252), and α-tubulin (1°Ab- 1:2000; anti-mouse 2°Ab- 1:10,000, 50 kDa) (Sigma-Aldrich, Burlington, MA, USA; T9026). The secondary antibodies anti-mouse-HRP (AP128P) and anti-rabbit-HRP (111-036-045) were purchased from Millipore Sigma and Jackson Immuno Research, respectively. Western blots were visualized using the SuperSignal Dura chemiluminescent detection kit (Pierce) and imaged using a FluorChem (Protein Simple, San Jose, CA, USA) imaging system and analyzed using FluorChem FC3 software (Protein Simple). Densitometric measurements were normalized against the expression levels of the ‘housekeeping’ protein α-tubulin and their respective WT controls.

### 2.5. Lipidomics

Cortical and hippocampal brain tissue was collected from a 6-month female cohort consisting with *n* = 3 mice per genotype. Lipid extraction was performed as previously described [46]. Briefly, frozen tissue samples were weighed, lyophilized, pulverized, and homogenized in 0.1× phosphate-buffered saline (PBS). Protein homogenates were quantified using a BCA protein assay kit (Pierce, Rockford, IL, USA). Lipids were extracted using a modified Bligh and Dyer extraction, as previously described [47,48] in the presence of internal standards which were added based on the total protein content of the sample. Multi-dimensional mass spectrometry (MDMS)-based shotgun lipidomics (MDMS-SL) was performed using electrospray ionization mass spectrometry (ESI/MS) to measure individual lipid molecular species. Instrumentation utilized a triple-quad mass spectrometer (Thermo Scientific TSQ Vantage, Waltham, MA, USA) equipped with a Nanomate device (Advion Bioscience Ltd., Ithaca, NY, USA) and Xcalibur system software was used as previously described [46,49,50]. Data processing including ion peak selection, baseline correction, data transfer, peak intensity comparison, ^13^C deisotoping, and quantitation were conducted using a custom programmed Microsoft Excel macro as previously described [50]. Relative concentrations of the total lipid class populations were calculated by summing individually detected analytes belonging to the class. The ANOVA analysis of the cortex and hippo were performed independently but to visualize the data and reflect variable localization of lipids across brain regions, the concentrations were normalized to the levels detected in the wildtype cortex for comparison.

### 2.6. Statistical Analysis

Data collection utilized instrumentation-specific software reported and compiled in Excel. Statistical analysis was performed using GraphPad Prism software (GraphPad Software, San Diego, CA, USA). Comparison of two groups were analyzed with an unpaired *t*-test with two-tailed *p*-value whereupon the definition of statistical significance is *p* ≤ 0.05 (*p* > 0.05 (ns), *p* ≤ 0.05 (*), *p* ≤ 0.01 (**), *p* ≤ 0.001 (***), and *p* ≤ 0.0001 (****)). Analysis of multiple groups utilized ANOVA analysis followed by Fisher’s PLSD post hoc test. *p*-values ≤ 0.05 were considered statistically significant (*p* > 0.05 (ns), *p* ≤ 0.05 (*), *p* ≤ 0.01 (**), *p* ≤ 0.001 (***), and *p* ≤ 0.0001 (****)). In comparisons of the multiple genotypes, correlations are symbolized as follows: * indicates significances from the WT, @ indicates significances from HIVgp120tg, and $ indicates significances from LTC4SKO mice.

## 3. Results

### 3.1. Quantitative Analysis of Mouse Brain Tissue via LC-MS/MS Shows an Increase in Eicosanoids in HIVgp120tg Mice, Particularly Products of the COX Pathway

**Figure 1:**
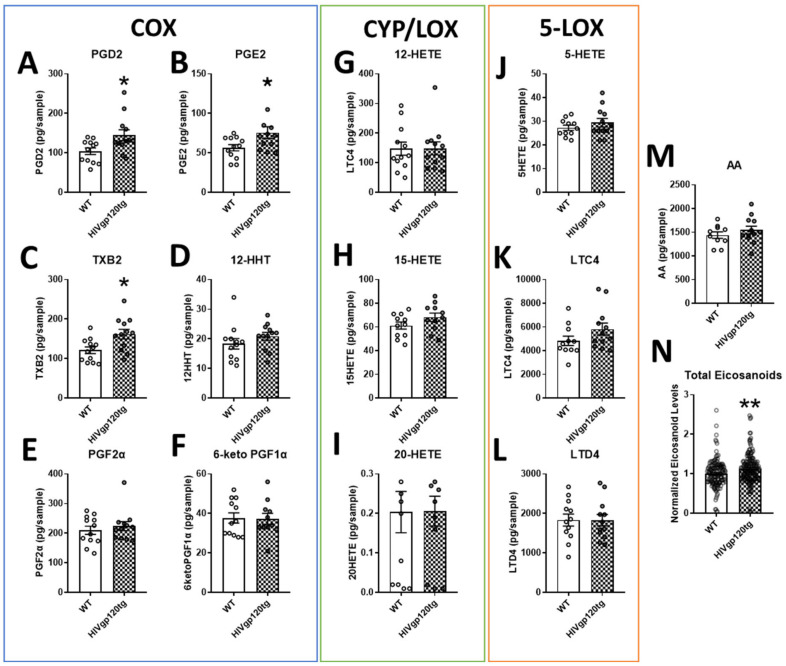
Quantification via LC-MS/MS of select eicosanoids in the brain extracts of wildtype (WT) and HIVgp120tg mice. COX pathway products outlined in blue (**A**–**F**), CYP/8-,12-,15-LOX products are outlined in green (**G**–**I**) and 5-LOX products are outlined in orange (**J**–**L**). The source material for the biosynthesis of eicosanoids, AA (**M**), and the total normalized values of all measured eicosanoid derivatives (**N**) are also shown. Values in graphs are mean ± s.e.m.; *n* = 12 (6 males and 6 females) per genotype; statistical analysis was performed using an unpaired *t*-test, * *p* ≤ 0.05, ** *p* ≤ 0.01 for difference to WT control. Individual data points are represented in the graphs by an ⚪ symbol. Abbreviations: COX, cyclooxygenase; CYP, cytochrome P450; LOX, lipoxygenase; AA, arachidonic acid; PG, prostaglandin; TX, thromboxane; HHT, hydroxyheptadecatrenoic acid; HETE, hydroxyeicosatetraenoic acid; LT, leukotriene/cysteinyl leukotriene (LTC4, LTD4).

Eicosanoid analytes measured in the brain extracts of HIVgp120tg mice can be categorized into the products of the three pathways of eicosanoid synthesis: COX (Figure 1A–F), CYP/8-,12-,15-LOX (Figure 1G–I), and 5-LOX (Figure 1J–L). Although the majority of byproducts of the CYP and LOX pathway trended upwards (LTC4 *p* = 0.14), findings were not statistically significant. Of the COX pathway, PGD2, PGE2, and TXB2 were significantly increased (*, *p* ≤ 0.05) in the brains of HIVgp120tg mice. Interestingly, free arachidonic acid was not found to be significantly increased despite the increase in its metabolites (Figure 1M). Normalized analyte levels of all eicosanoid metabolites were significantly heightened (**, *p* ≤ 0.01) in the HIVgp120tg when compared to the levels detected in WT mice (Figure 1N). Of note, the analysis of eicosanoids did not detect any sex-dependent differences.

### 3.2. Gene Expression Signatures of HIVgp120tg and LTC4S Knockout Mice Reveal Fundamental Sexual Dimorphism in Eicosanoid Pathway-Associated Genes

**Figure 2:**
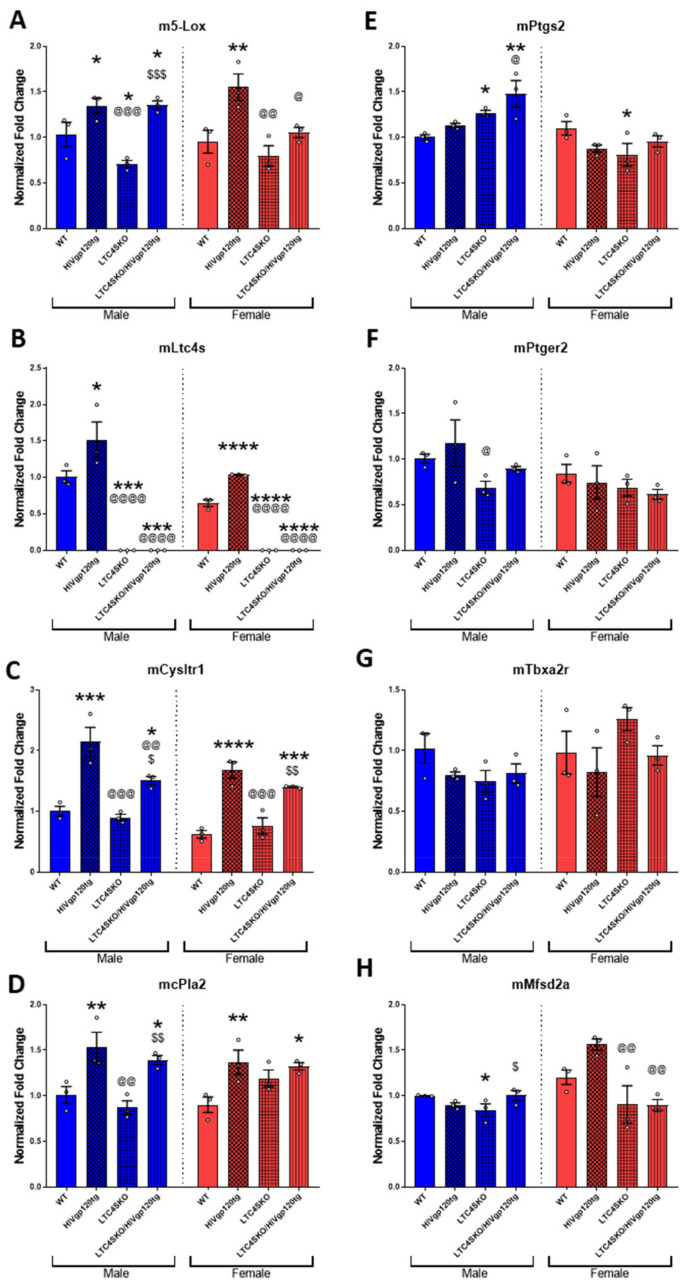
Gene expression levels quantified via qRT-PCR of eicosanoid pathway transcripts and lipid-associated proteins exhibit sexual dimorphism. LOX pathway-associated genes, m5-Lox (**A**), mLtc4s (**B**) and mCysLtr1 (**C**); eicosanoid cascade initiator, mcPla2 (**D**); COX pathway-associated genes, mPtgs2 (**E**), mPtger2 (**F**), and mTbxa2r (**G**); blood–brain barrier (BBB) membrane-associated fatty acid transport protein, mMfsd2a (**H**). Male (blue) and female (red) data sets are shown separated by a dashed line. Values in graphs are mean ± s.e.m.; *n* = 3 per genotype, cortex tissue, 12 months of age; ANOVA followed by Fisher’s PLSD post hoc test for males and females were analyzed independently while normalized to their respective WT controls while graphs are shown above as normalized to male WT controls to illustrate gender differences. Individual data points are represented in the graphs by an ⚪ symbol. The *p*-value level of significance is as follows: * *p* ≤ 0.05, ** *p* ≤ 0.01, *** *p* ≤ 0.001, **** *p* ≤ 0.0001, $ *p* ≤ 0.05, $$ *p* ≤ 0.01, $$$ *p* ≤ 0.001, @ *p* ≤ 0.05, @@ *p* ≤ 0.01, @@@ *p* ≤ 0.001, @@@@ *p* ≤ 0.0001—while *p*-value significance correlations are symbolized as follows: * indicates significances from the WT, @ indicates significances from HIVgp120tg, and $ indicates significances from LTC4SKO mice.

The major synthetic enzyme for the 5-LOX pathway, 5-Lox, the enzyme responsible for the production of all CysLTs, LTC4S, and the CysLT receptor most associated with inflammation, cysteinyl leukotriene receptor 1 (CysLTR1), as well as the enzyme that initiates the eicosanoid cascade as a whole, cPla2, are all significantly upregulated in mRNA isolated from the cortices of HIVgp120tg mice (male 5-Lox, Ltc4s: *, *p* ≤ 0.05; female 5-Lox: **, *p* ≤ 0.01; male Cysltr1: ***, *p* ≤ 0.001; female Ltc4s, Cysltr1: ****, *p* ≤ 0.0001, cPla2: *, *p* ≤ 0.05), irrespective of sex (Figure 2A–D). However, male LTC4SKO significantly expressed lower levels of 5-Lox (*, *p* ≤ 0.05), while the HIVgp120-induced upregulation (*, *p* ≤ 0.05) was unaltered in LTC4SKO/HIVgp120tg mice (*, *p* ≤ 0.05) (Figure 2A). In comparison, female mice had no significant downregulation of 5-Lox in the absence of LTC4S, while LTC4SKO/HIVgp120tg had significantly lower levels of 5-Lox than HIVgp120tg mice (@, *p* ≤ 0.05). Expression levels of Cysltr1 in LTC4SKO/HIVgp120tg mice exhibited a significant decrease in males (@@, *p* ≤ 0.01) compared to HIVgp120tg mice (Figure 2C).

Expression of cPla2, which begins the eicosanoid pathway with the release of cell membrane phospholipid-associated AA, is also heightened in both male (**, *p* ≤ 0.01) and female (**, *p* ≤ 0.01) mice by the presence of the HIVgp120 transgene. Interestingly, male LTC4SKO mice had a significant increase in cPla2 expression with the expression of the HIVgp120 transgene ($$, *p* ≤ 0.01) which did not significantly differ in female LTC4SKO mice (Figure 2D).

Unlike the leukotriene-associated genes, the COX pathway gene expression did not follow a general rising trend due to HIVgp120 transgene expression. The inflammatory COX pathway enzyme, prostaglandin-endoperoxide synthase 2 (Ptgs2 also COX2) had substantial sex differences, particularly in LTC4SKO animals. The LTC4SKO decreased the levels of Ptgs2 in female mice alone (*, *p* ≤ 0.05). While in male mice, LTC4SKO led to a significant increase (*, *p* ≤ 0.05) which was further increased in the LTC4SKO/HIVgp120tg mice (**, *p* ≤ 0.01) (Figure 2E). Prostaglandin E2 receptor 2 (Ptger2, also known as EP2) was significantly decreased in the male LTC4SKO mice compared to the HIVgp120tg mice (@, *p* ≤ 0.05) (Figure 2F). The gene expression of thromboxane A2 receptor (Tbxa2r) in male mice was reduced by the absence of LTC4S but did not reach statistical significance (*p* = 0.0580). In contrast, female LTC4SKO had trending higher levels of Tbxa2r than those found in their HIVgp120tg counterparts, though again, not reaching statistical significance (*p* = 0.0688) (Figure 2G).

The blood–brain barrier (BBB) membrane-associated fatty acid transport protein, major facilitator superfamily domain-containing protein 2a (Mfsd2a), exhibited strong sexual dimorphic effects in the HIVgp120tg mice (Figure 2H). Mfsd2a in HIVgp120tg females trended higher (*p* = 0.0643) for this BBB-associated gene. LTC4SKO decreased Mfsd2a levels in male (*, *p* ≤ 0.01). However, HIVgp120 expression increased the levels of the gene in LTC4SKO/HIVgp120tg males compared to LTC4SKO ($, *p* ≤ 0.01) alone. However, in LTC4SKO/HIVgp120tg females, Mfsd2a levels were suppressed (@@, *p* ≤ 0.01) and prevented HIVgp120-induced increases in expression (@@, *p* ≤ 0.01) compared to HIVgp120tg levels alone.

### 3.3. Western Blot Reveals Sexually Dimorphic Suppression of MAPK Signaling Pathway by Genetic Knockout of LTC4S

**Figure 3:**
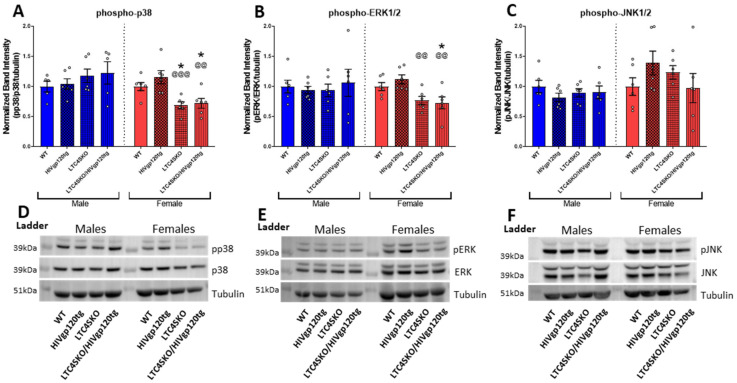
MAPK signaling pathway in female LTC4SKO mice shows the reduced activity levels of p38 and ERK in protein brain lysates. The quantification of Western blot densitometries of: phosphorylated p38 MAPK (pp38)/total p38/tubulin (p38) (**A**); phosphorylated ERK1/2 (pERK)/total ERK1/2/tubulin (ERK) (**B**); and phosphorylated JNK1/2 (pJNK)/total JNK1/2/tubulin (JNK) (**C**) all normalized to their respective wild type control. Representative images of the blots are shown with male and female samples separated by a protein ladder lane are for p38 (**D**), ERK1/2 (**E**), and JNK1/2 (**F**). Male (blue) and female (red) data sets are shown, separated by a dashed line. Values in graphs are mean ± s.e.m.; *n* = 4–6 per genotype for both male and female data sets, 12 months of age. Individual data points are represented in the graphs by an ⚪ symbol. ANOVA and Fisher’s PLSD post hoc test for males and females were analyzed independently while normalized to their respective WT controls; *p*-value level of significance is as follows: * *p* ≤ 0.05, @@ *p* ≤ 0.01, @@@ *p* ≤ 0.001—while the *p*-value significance correlations are symbolized as follows: * indicates significances from the WT, @ indicates significances from HIVgp120tg.

Protein lysates from both males and females were probed for three well-known mammalian mitogen-activated protein kinase (MAPK) pathways to assess activity: the extracellular signal-regulated protein kinase 1/2 (ERK1/2), c-Jun N-terminal kinase 1/2 (JNK1/2), and the p38 MAPK (Figure 3A–F). Our lab has previously reported the importance of MAPK signaling in HIVgp120-induced neurotoxicity [51,52,53]. The quantification of Western blot densitometries shows reduced levels of phosphorylated p38 MAPK and ERK1/2 in LTC4SKO/HIVgp120tg female brains only (Figure 3A,D). Male mice showed little to no alteration in any MAPK signaling following LTC4SKO in WT and HIVgp120tg mice. Female LTC4SKO mice had significantly lower levels of p38 activity (*, *p* ≤ 0.05) which remained significantly lower despite the expression of the HIVgp120 transgene (*, *p* ≤ 0.05). The levels of phosphorylated p38 in LTC4SKO/HIVgp120tg mice were also significantly lower than those detected in WT (*, *p* ≤ 0.05) and in HIVgp120tg mice (@@, *p* ≤ 0.01). Phosphorylated ERK1/2 showed similar trends with female LTC4SKO/HIVgp120tg mice having decreased the levels of ERK1/2 activity compared to both their WT controls (*, *p* ≤ 0.05) and their HIVgp120tg counterparts (@@, *p* ≤ 0.01) (Figure 3B,E). Phosphorylated JNK1/2 in LTC4SKO/HIVgp120tg female mice also showed similar trends but did not reach a level of statistical significance (Figure 3C,F).

### 3.4. Shotgun Lipidomics of Both Cortical and Hippocampal Extracts of Female Mice Show Alterations in Lipid Populations Due to Both HIVgp120 Transgene and LTC4S Knockout

**Figure 4:**
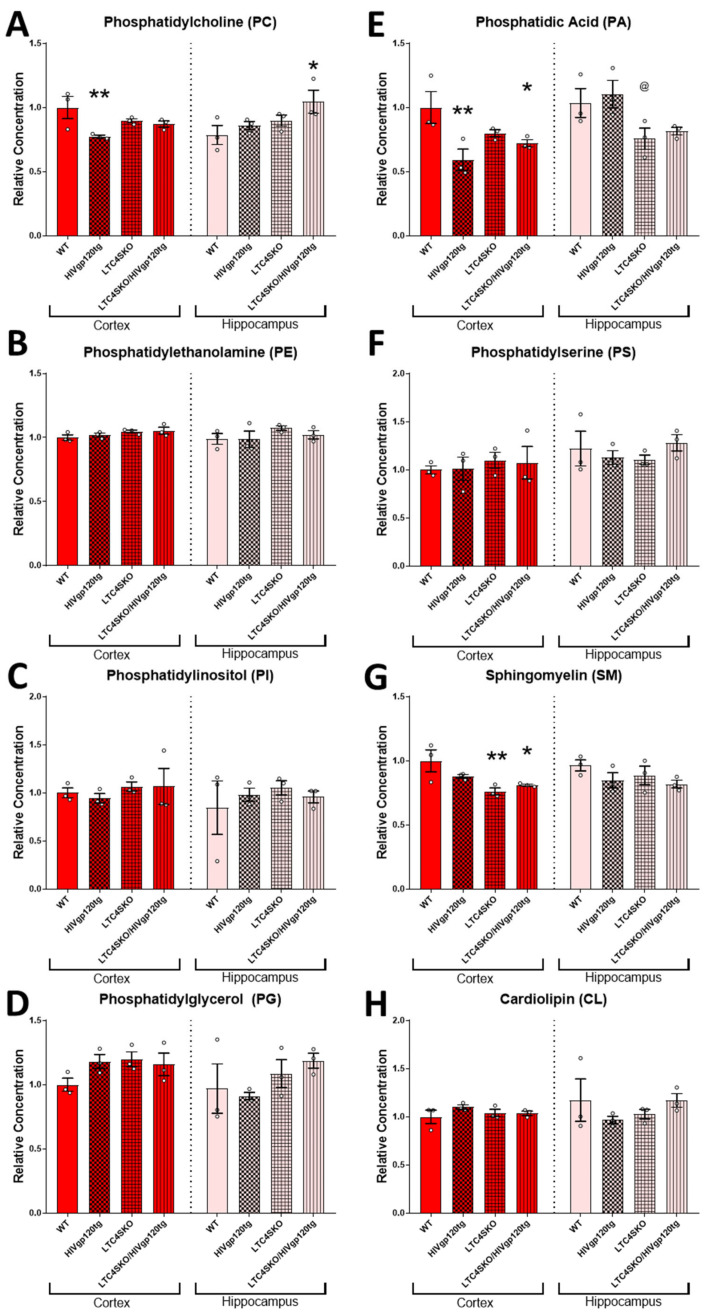

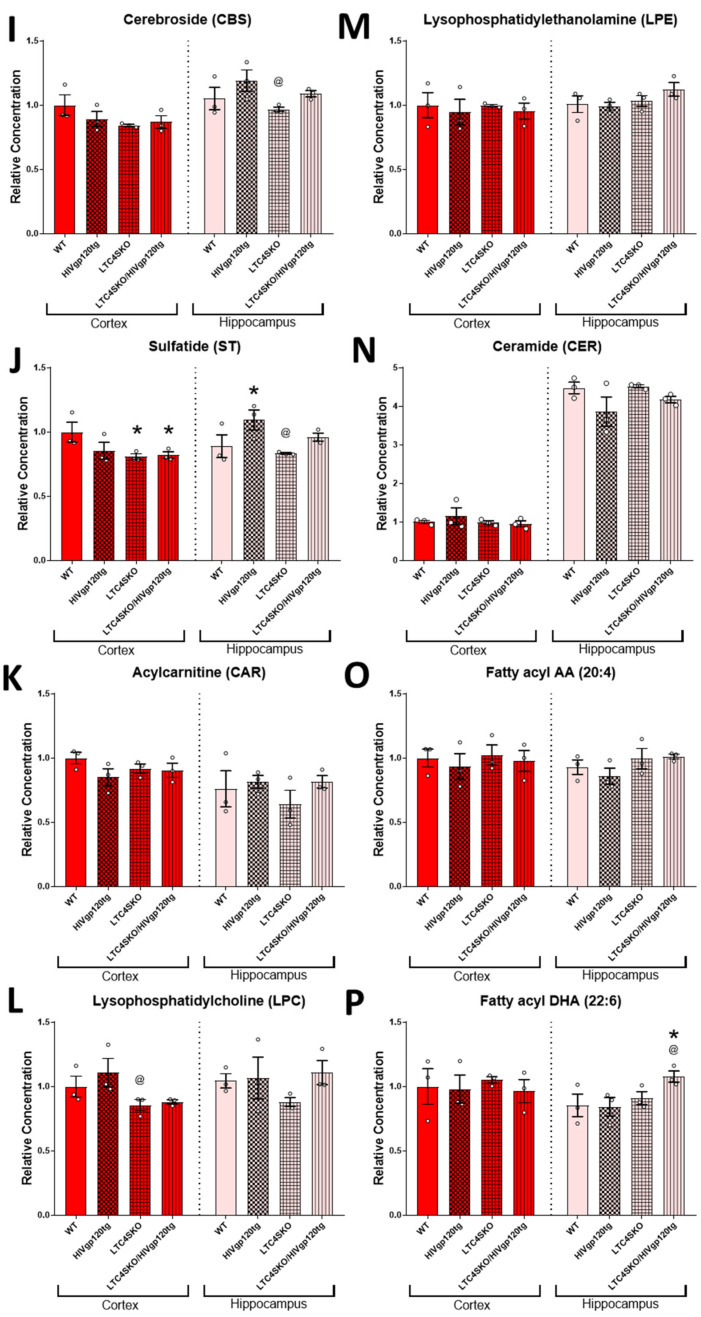
(Previous two pages) Total lipid class populations in cortex and hippocampal tissue extracts show that HIVgp120tg- and LTC4SKO-induced alterations have differential and regional effects. Individual analytes of lipid classes measured through multi-dimensional mass spectrometry-based shotgun lipidomics (MDMS-SL) were summed to equate the representative population for each lipid class. Lipid classes are broadly organized by functional roles: membrane components (**A**–**J**), energy storage and metabolism (**K**), followed by signaling lipids (**J**–**N**). Additionally, fatty acyl (FA) containing lipids across all classes were quantified for arachidonic acid 20:4 FA (**O**) and docosahexaenoic acid 22:6 FA (**P**). Bar graphs displaying cortical lipids (red) and hippocampal lipids (pink) are shown separated by a dashed line. Values in the graphs are mean ± s.e.m.; *n* = 3 per genotype, female, 6 months of age. Individual data points are represented in the graphs by an ⚪ symbol. ANOVA followed by Fisher’s PLSD post hoc test for cortex lipids and hippocampus lipids were independently analyzed and normalized to their respective WT controls—while the graphs shown above are normalized to cortex WT controls levels to illustrate regional differences. *p*-value level of significance is as follows: * *p* ≤ 0.05, ** *p* ≤ 0.01, @ *p* ≤ 0.05—while *p*-value significance correlations are symbolized as follows: * indicates the significances from the WT, @ indicates significances from HIVgp120tg.

The shotgun lipidomics focused on brain samples from females since they experienced more significant alterations of the eicosanoid pathway in association with the LTC4SKO. Tissues were collected from a 6-month-old cohort as, at this age, the HIVgp120tg mice exhibit early stages of neuropathology but have yet to show significant behavioral impairment, a status corresponding to many slow progressing NeuroHIV patients or HIV patients on cART [4,7,41,54]. Lipids from the following classes were assessed via MDMS-SL: phosphatidylcholine (PC), phosphatidylethanolamine (PE), phosphatidylinositol (PI), phosphatidylglycerol (PG), phosphatidic acid (PA), phosphatidylserine (PS), sphingomyelin (SM), cardiolipin (CL), cerebroside (CBS), sulfatide (ST), acylcarnitine (CAR), lysophosphatidylcholine (LPC), lysophosphatidylethanolamine (LPE), and ceramide (CER). Additionally, the fatty acid (FA) containing lipids across all classes were quantified for n-6 PUFA arachidonic acid 20:4 FA (AA, 20:4) and n-3 PUFA docosahexaenoic acid 22:6 FA (DHA, 22:6) derivatives to extrapolate bioavailability (Figure 4A–P).

HIVgp120 transgene expression led to decreased cortical PC (**, *p* ≤ 0.01) and PA (**, *p* ≤ 0.01) levels and increased hippocampal ST (*, *p* ≤ 0.05) levels compared to WT controls (Figure 4A,E,J). Genetic ablation of LTC4S lowered cortical SM (**, *p* ≤ 0.01) and ST (*, *p* ≤ 0.05) concentrations compared to WT controls (Figure 4G,J). The LTC4SKO in HIVgp120tg mice led to decreases in the cortical levels of lipids’ PA (*, *p* ≤ 0.05), SM (*, *p* ≤ 0.05), and ST (*, *p* ≤ 0.05) (Figure 4E,G,J) and increased the hippocampal levels of PC (*, *p* ≤ 0.05) and fatty acyl DHA 22:6 (*, *p* ≤ 0.05) compared to WT controls (Figure 4A,P).

**Figure 5:**
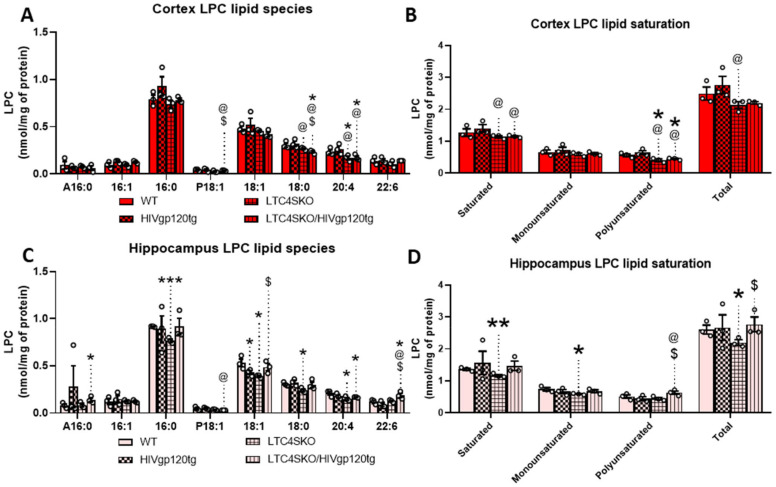
Lysophosphatidylcholine (LPC) lipid species levels show differential fatty acid composition in the cortex and hippocampus of female mice as a result of LTC4S knockout. Analysis of the subspecies within the LPC lipid class of the lipid extracts from both cortex (**A**,**B**) and hippocampus (**C**,**D**) were quantified by MDMS-SL. Individual LPC species with masses above 0.05 nm/mg were graphed for cortex (**A**) and hippocampus (**C**). Total masses of saturated, monounsaturated, polyunsaturated, and total LPCs with masses above 0.05 nm/mg were quantified and plotted for cortex (**B**) and hippocampus (**D**). Bar graphs displaying cortical lipids (red) and hippocampal lipids (pink) are shown separated by a dashed line. Values in graphs are mean ± s.e.m.; *n* = 3, female, 6 months of age. Individual data points are represented in the graphs by an ⚪ symbol. ANOVA followed by Fisher’s PLSD post hoc test for cortex lipids and hippocampus lipids were independently analyzed for each fatty acid subspecies—while graphs shown above are reflected on a single *x* axis to illustrate intraspecies differences. The *p*-value level of significance is as follows: * *p* ≤ 0.05, ** *p* ≤ 0.01, *** *p* ≤ 0.001, @ *p* ≤ 0.05, $ *p* ≤ 0.05—while *p*-value significance correlations are symbolized as follows: * indicates significances from the WT, @ indicates significances from HIVgp120tg, and $ indicates significances from LTC4SKO mice.

Further analysis of the LPC lipid class shows alterations in several individual species. Of the LPCs, the most abundant species found in our analysis were LPC palmitate (16:0), LPC oleate (18:1), LPC stearate (18:0), LPC eicosatetraenoate (20:4), LPC docosa-hexaenoate (22:6) (Figure 5A). Lower abundance species including linkages 16:1, A16:0, and P18:1 were also included as they met the minimum threshold for analysis (≥0.05 nm/mg). In the cortex, LTC4SKO led to lower levels of LPC 20:4 (*, @, *p* ≤ 0.05) which were sustained in the LTC4SKO/HIVgp120tg (*, @, *p* ≤ 0.05) animals (Figure 5A). Additionally, LPC 18:0 in cortices of LTC4SKO/HIVgp120tg animals was found to be significantly lower than those detected in both WT (*, *p* ≤ 0.05) and HIVgp120tg mice (@, *p* ≤ 0.05). Lipid saturation in LPC in LTC4SKO/HIVgp120tg also led to decreases in both saturated (@, *p* ≤ 0.05) and polyunsaturated (*, @, *p* ≤ 0.05) fatty acid linkages (Figure 5B). In the hippocampus, LTC4SKO led to lower levels of LPC 16:0 (***, *p* ≤ 0.001), LPC 18:1 (*, *p* ≤ 0.05), LPC 18:0 (*, *p* ≤ 0.05), and LPC 20:4 (*, *p* ≤ 0.05) (Figure 5C). The decrease in LPC 20:4 was sustained (*, *p* ≤ 0.05) in LTC4SKO/HIVgp120tg mice. Additionally, LTC4SKO/HIVgp120tg mice showed an increase in LPC 22:6 compared to any of the other genotypes (*, @, $, *p* ≤ 0.05). The fatty acid lipid saturation of LPC in the hippocampus showed a decrease in saturated (**, *p* ≤ 0.01) and monounsaturated (*, *p* ≤ 0.05) levels in the LTC4SKO compared to WT controls (Figure 5D). Unlike the cortex, the HIVgp120 expression in the LTC4SKO mice increased the levels of polyunsaturated LPC ($, *p* ≤ 0.05) in the hippocampus.

## 4. Discussion

Classically, eicosanoids play a role in the acute immune response, activating a cascade of pro-inflammatory effects. However, it is now known that eicosanoids play an important role in anti-inflammatory processes and the resolution of inflammation. Their role in either the pro- or anti- inflammatory side appears to be dictated by the molecular milieu as well as the presence of specific receptors with different signal transduction pathways [55,56,57,58]. The complete resolution of inflammation would ideally dampen eicosanoid release. However, the persistent activation of the pathway is a sign of chronic inflammation [57,59,60] to the detriment of neuronal health and cognitive function [61]. HIVgp120tg mice exhibit chronic inflammation markers in the brain in the form of astrocytosis and increased numbers of activated microglia [39] with increased levels of pro-inflammatory and neurotoxic elements [41]. We found that HIVgp120tg mice exhibit a heightened production of COX pathway derivatives PGD2, PGE2, and TXB2 (*, *p* ≤ 0.05) and an overall increase in all measured metabolites of the AA cascade (**, *p* ≤ 0.01). Imbalances in eicosanoid synthesis and increases in COX activity were extensively reported about for Alzheimer’s disease (AD) [61,62]. However, despite evidence that the inhibition of COX significantly protects against the development of AD, clinical trials have failed to demonstrate either an improvement in symptoms or the prevention of the disease [62]. This may be due to the fact that the pharmacological targeting of the eicosanoid cascade often results in broad alterations across the entire AA metabolic network [63] with a blockade of the COX pathway often resulting in a shift towards the 5-LOX pathway [64,65]. Similarly, the inhibition of the 5-LOX pathway tends to cause available AA to shunt into the COX pathway and simultaneously prevent the formation of 12/15 HETE-derived lipoxins (LXs) which are critical for the resolution of inflammation [55,66,67]. Studies into the inhibition of more downstream COX and LOX enzymes have found a less considerable effect on AA cascade flux [63]. Thus, our lab was interested in the effect of the genetic depletion of LTC4S, a downstream target of the 5-LOX pathway, as a potential therapeutic strategy for chronic neuroinflammation in the HIVgp120tg mouse model of NeuroHIV.

While the analysis of eicosanoids did not detect any sex-dependent differences, transcriptomic analysis revealed the sex differential expression of the AA cascade as a result of both HIVgp120 transgene expression as well as LTC4SKO. Regarding eicosanoid pathway alterations, females experienced more significant alterations as a result of LTC4SKO. For LOX pathway genes, LTC4SKO mice led to a reduction in the gp120-induced expression of 5-LOX in female mice only (@, *p* ≤ 0.05)), suggesting a possible feedback mechanism for 5-LOX that only exists in female mice. Interestingly, the same knockout resulted in similar reductions in CysLTR1 for males (@@, *p* ≤ 0.01) with a downward trend in females (*p* = 0.0851). For cPla2 expression, the knockout of LTC4S appears to raise the baseline transcript levels of cPla2 in female mice alone (*p* = 0.0610); however, HIVgp120 transgene-induced increases in cPla2 were unaltered. COX2 gene expression varied significantly between the sexes. LTC4SKO males had significant increases in Ptgs2 (*, *p* ≤ 0.05), which further increased upon the expression of the HIVgp120 gene (**, *p* ≤ 0.01; @, *p* ≤ 0.05). In females, LTC4S caused a decrease in the gene expression of Ptgs2 enzyme (*, *p* ≤ 0.05), but this decrease was not sustained in LTC4SKO/HIVgp120tg mice. Interestingly, the expression of Mfsd2a, a fatty acid transport protein responsible for carrying long-chain fatty acids esterified to LPC across the BBB, had significant sexual dimorphic trends in LTC4SKO/HIVgp120tg mice. Quantification shows that LTC4SKO decreased the levels of the Mfsd2a gene in males (*, *p* ≤ 0.05) but this suppression was not sustained in the LTC4SKO/HIVgp120tg mice ($, *p* ≤ 0.05). However, the female levels of the Mfsd2a were suppressed in both the LTC4SKO and the LTC4SKO/HIVgp120tg mice compared to the HIVgp120tg alone (@@, *p* ≤ 0.01). Mfsd2a is a critical component for the health and function of the BBB, and the genetic knockdown of the protein leads to leaky BBB from embryonic stages through adulthood [68]. Furthermore, Mfsd2a is the major transporter for DHA uptake in the brain in addition to being an omega-3 fatty acid that is critical in brain development and in aging for the preservation of neuronal health and cognitive function [69,70,71], as well as in resolution of neuroinflammation by DHA-derived lipid mediators such as resolvins and neuroprotectins [72,73,74].

Seeking further for the neuroinflammatory signaling pathways underlying HIVgp120tg-induced damage, the Western blot of the MAPK network was performed. In general, phosphorylation by ERK and p38 MAPK increases the enzymatic activity of cPla2 and stimulates the release of arachidonic acid [75,76]. Overall, we found that the differences in MAPK activity in HIVgp120tg animals were not statistically significant between the male and female groups. However, LTC4SKO in the HIVgp120tg caused significant drops in phosphorylated p38 (@@, *p* ≤ 0.01) and ERK1/2 (@@, *p* ≤ 0.01) of female mice only.

In order to investigate whether these changes might alter the lipidomic profile in the brain, MDMS-SL was performed on female cortical and hippocampal lipid extracts. Alterations in the lipid species of both brain regions occurred, though most were found in the cortex. Two classes of lipids were found to be lowered by the expression of the HIVgp120 transgene and by the knockout of LTC4S: PC (**, *p* ≤ 0.01) and PA (**, *p* ≤ 0.01); and SM (**, *p* ≤ 0.01) and ST (*, *p* ≤ 0.05), respectively. These four classes are broadly known as membrane structural components and alterations in these species could alter membrane permeability/fluidity, interactions among membrane proteins such as ion channels, and the microenvironment in cell-to-cell communication networks [77]. The HIVgp120-induced reduction in PC has also been noted in the late stages of AD and AD mouse models [78,79,80]. Alterations in PA metabolism were also implicated in a number of different neurological diseases [81] and appear to be critical for the exocytosis and endocytosis of synaptic vesicles [82]. The absence of LTC4S reduced levels of ST, which is a marker of myelin sheaths, and substantial losses of which have been reported in very early stages of AD [83]. Levels of SM were also reduced by LTC4SKO, which has been reported in some studies to have also been either reduced or increased in AD brains, both of which impact Aβ pathology [84,85,86]. SM supports brain myelination and is thus essential for brain development and cognition [87] and is altered in neurodegeneration [88,89]. Reductions in both SM and ST in LTC4SKO mice may be indicative of aberrant sphingolipid metabolism which has been linked to a number of neurodegenerative processes such as AD, PD, and HANDs [90,91]. Several studies of patients with HIV have found that accumulations of sphingomyelin and ceramide occur in brain tissue and cerebrospinal fluid associated with cognitive impairment [92,93,94,95]. Moreover, cell culture experiments showed that pharmaceutical intervention into this sphingolipid increase can protect neurons from HIV-neurotoxic proteins [92]. It has been proposed that the progressive cognitive impairment characterized by accumulations in multiple sphingomyelin species leads to the perturbation of endolysomal function due to sequestration of excess sphingolipids [90]. Interestingly, despite the fact that cortical qPCR results indicate lowered levels of DHA transport protein Mfsd2a in LTC4SKO females, the levels of fatty acyl DHA in the lipid population were not significantly altered across the genotypes. Furthermore, DHA-bound lipids were significantly increased in the hippocampus of LTC4SKO/HIVgp120tg animals (*, *p* ≤ 0.05). This increase in DHA 22:6 FA in hippocampal tissue may be due to regionally constrained increases in uptake obtained from circulation, since the synthesis of DHA in the brain is negligible [96].

Plasma fatty acids can only enter the brain in two forms: non-esterified free fatty acids and esterified in the form of LPC. Furthermore, the fatty acids bound to LPCs cross the BBB more efficiently than their unbound counterparts [97,98,99,100,101]. Thus, the analysis of the LPC fatty acid derivatives provide a useful window by which to view the available fatty acid supply to the brain. Measurements of the monoacylglycerol LPC fatty acid species revealed that AA-containing-LPC levels decreased in LTC4SKO (*, *p* ≤ 0.05) and LTC4SKO/HIVgp120tg (*, *p* ≤ 0.05) mice in both the cortex and the hippocampus. These lowered levels would, in theory, limit the availability of AA and thus the source of eicosanoid biosynthesis. The DHA containing LPC levels corresponded to the total DHA-containing lipid population, with significant increases in the hippocampus of LTC4SKO/HIVgp120tg mice (*, *p* ≤ 0.05). The presence of DHA in the brain has been shown to be extremely beneficial during development and in a multitude of neurological disorders including neuroinflammation due to their role as source material for the generation of anti-inflammatory resolvins and neuroprotectin [69,70,71,72,73,74]. The lipid saturation of LPC saw the lowering of saturated (@, *p* ≤ 0.05) and polyunsaturated (@, *p* ≤ 0.05) linkages following LTC4SKO in HIVgp120tg animals. Hippocampus lipid extracts showed lowered levels of saturated (**, *p* ≤ 0.01) and monounsaturated acids in LTC4SKO (*, *p* ≤ 0.05) compared to the control, although this decrease was abolished by HIVgp120 expression. Conversely, polyunsaturated LPC increased in the hippocampus of LTC4SKO/HIVgp120tg mice (@, *p* ≤ 0.05) compared to HIVgp120tg mice with the WT alleles of LTC4S. The level of unsaturation (double bonds) in fatty acid side chains alters the three-dimensional structure of lipids and could exhibit different impacts on neuroinflammation and oxidative damage [102]. Shotgun lipidomics performed on two AD mouse models revealed APP overexpression and high oligomeric Aβ content leads to significant increases in the saturated, monounsaturated, and polyunsaturated LPC fatty acids. These increases arise in conjunction with heightened levels of phosphorylated ERK1/2 and p38 MAPK [46]. In our model, LTC4SKO decreases these pathological markers, making it appealing as a strategy for possible pharmacological targeting. In fact, current research is being performed on targeting CysLT production and signaling receptors in a number of CNS diseases including brain injury, AD, and PD [103].

## 5. Conclusions

Eicosanoid synthesis is a delicate balancing act which governs the cellular immune response through the selective production of pro- and anti-inflammatory products. Consequently, attempts to target one branch such as the COX pathway often lead to the shunting of AA into the 5-LOX pathway. Thus, the pharmaceutical targeting of a single route for these dual pro-inflammatory paths often produces undesirable side effects [63,65,104,105]. Therefore, we attempted the genetic depletion of a downstream enzyme in the 5-LOX pathway, LTC4S, responsible for the production of CysLTs. While eicosanoid levels lacked sex-dependence, our findings show that significant sexual dimorphism results from the genetic ablation of this LTC4S. The analysis of AA cascade-related transcripts shows inherent differences in the baseline expression of both enzymes and receptors for eicosanoid signaling. Additionally, LTC4SKO led to the selective suppression of MAPK signaling activity in female mice only. Shotgun lipidomics confirms that LTC4S knockout in females also leads to alterations in the lipid composition in the brain.

Gender differences in androgen-mediated eicosanoid production are well defined—with males showing more emphasis on COX/prostaglandins while females have inherently more LOX/leukotriene formation [106]. Though the quantification of the eicosanoid lipid mediators in WT and HIVgp120tg mice did not reveal sexually dimorphic or age-related differences in the concentrations of PG or LT in the brains, the transcriptional analysis of mice at 12 months of age revealed differences in gene expression levels. It should be noted that measurements of the gene expression levels at 6 months of age were also sexually dimorphic but had sometimes opposing trends in baseline levels (Figure S1), possibly indicative of the age and hormonal status of the animals. Measurements of the body and brain masses of the collected mice also indicate both sexual dimorphism (Figure S2) and age-related alterations (Figure S3). Several studies have observed that females not only produce higher levels of LTs but also respond to LT modifiers with higher efficacy than males [107,108]. Thus, it cannot be discounted that the pharmaceutical targeting of LTC4S and its downstream receptors could have sexually dimorphic benefits in females alone. The present study provides a rationale for a future lipidomics study including males.

Future studies of the feasibility of the pharmacological modulation of eicosanoid signaling or arachidonic acid cascade for the treatment or prevention of neurodegeneration must take into account possible personalized medicine regimes for patients on the basis of these gender differences. Reports from UNAIDS estimate that, currently, females account for more than half of all people living with HIV (53%) [1]. Among the risk factors for HANDs, biological sex consistently remains associated with higher odds of HANDs [109]. Consequently, women with HIV may be more vulnerable to the development of impairment and demonstrate greater impairment than in males [110,111,112,113]. Thus, this at-risk group may benefit enormously from the addition of an anti-inflammatory 5-LOX signaling mediator to their therapeutic drug regimens to protect or prevent HIV-mediated neurotoxicity and neurodegeneration.

## Figures and Tables

**Table 1:** Primers for qRT-PCR.

Gene	Gen Bank	Primer Sequence (5′-3′)
mLtc4s	NM_008521.2	Fwd: TCG TGG GAG TTC TGT TGC AA Rev: GAA AGC CCT TCG TGC AGA GA
mCysltr1	NM_021476.5	Fwd: GGA AGG CTG ATT TCT CAT GG Rev: GGA ACT GAA AAT CTG ACG ACA TC
mcPla2	NM_008869.4	Fwd: CTG CAA GGC CGA GTG ACA Rev: TTC GCC CAC TTC TCT GCA A
mPtgs2	NM_011198.4	Fwd: GGC GCA GTT TAT GTT GTC TGT Rev: CAA GAC AGA TCA TAA GCG AGG A
mTau	NM_001038609.3	Fwd: TGA GGG ACT AGG GCA GCT AA Rev: CTG CCT TCC TCA CCT CTG TC
mMfsd2a	NM_029662.2	Fwd: TTG CTA TGC AGT TGG AGG GG Rev: CGC ACG CCT AGG ATC AGA AT
mGAPDH	NM_001289726.1	Fwd: AGGTCGGTGTGAACGGATTTG Rev: TGTAGACCATGTAGTTGAGGTCA

## Data Availability

The data presented in this study are either available in Supplementary Materials or upon request from the corresponding author.

## Supplementary Materials

The following supporting information can be downloaded at: https://www.mdpi.com/article/10.3390/cells11132123/s1, Figure S1. Age-related and sexually dimorphic gene expression levels of eicosanoid pathway transcripts in cortex mRNA of HIVgp120tg mice; Figure S2. Brain and body weight alterations resulting from HIVgp120tg and LTC4SKO at 12 months of age; Figure S3. Brain and body weight alterations resulting from HIVgp120tg and LTC4SKO among females harvested for shotgun lipidomics at 6 months of age; Table S1. The mass transitions and collision energies used in LCMS/MS data analysis.
