# Supplementary material for: Arachidonic Acid Cascade and Eicosanoid Production Are Elevated While LTC4 Synthase Modulates the Lipidomics Profile in the Brain of the HIVgp120-Transgenic Mouse Model of NeuroHIV
Source: Cells. 2022 Jul 5;11(13):2123. doi: 10.3390/cells11132123 (PMC9265961; doi:10.3390/cells11132123)
Supplement: Supplementary file 1 [file cells-11-02123-s001.zip › cells-1758467-supplementary.pdf]

Supplementary Materials:

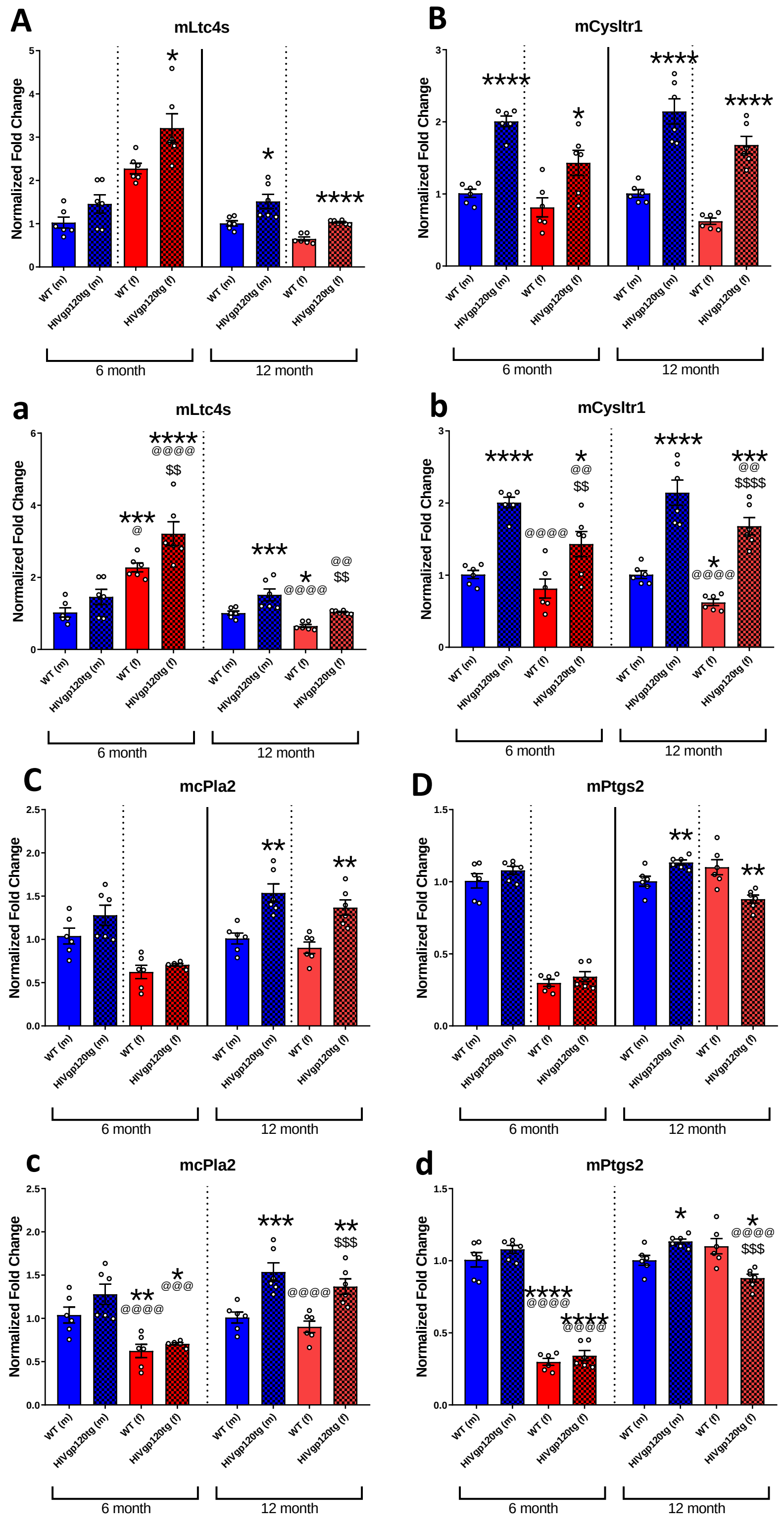

Figure S1. Age-related and sexually dimorphic gene expression levels of eicosanoid pathway transcripts in cortex mRNA of HIVgp120tg mice. LOX pathway associated genes, mLtc4s (**A, a**) and mCysLtr1 (**B, b**); eicosanoid cascade initiator, mcPla2 (**C, c**); COX pathway associated genes, mPtgs2 (**D, d**). Male (blue) and female (red) data sets (**A-D**) in 6 months of age (left side) and 12 months of age (right side) shown separated by a solid black line, while separate sexes within both age groups were further segmented with a dashed black line. Comparison within the two age groups (**a-d**) were separated by a dashed black line. Values in graphs are mean ± s.e.m.; n = 3 per genotype. Individual data points are represented in the graphs by an ◯ symbol. Statistical analysis between WT and HIVgp120tg mice (**A-D**) was separately performed for each sex and each age group using an unpaired t-test, * $p \leq 0.05$, ** $p \leq 0.01$, *** $p \leq 0.001$, **** $p \leq 0.0001$ for difference to their respective WT control. For comparison between sexes of the same age group (**a-d**), ANOVA followed by Fisher's PLSD post hoc test were analyzed while normalized to male WT controls of the same age group. Graphs are shown on the same axis to illustrate both sex and age-related differences. The *p*-value level of significance is as follows: * $p \leq 0.05$, ** $p \leq 0.01$, *** $p \leq 0.001$, **** $p \leq 0.0001$, @ $p \leq 0.05$, @@ $p \leq 0.01$, @@@ $p \leq 0.001$, @@@@ $p \leq 0.0001$, $$ $p \leq 0.01$, $$$ $p \leq 0.001$, $$$$ $p \leq 0.0001$; while *p*-value significance correlations are symbolized as follow: * indicates significances from the WT, @ indicates significances from HIVgp120tg, and $ indicates significances from LTC4SKO mice.

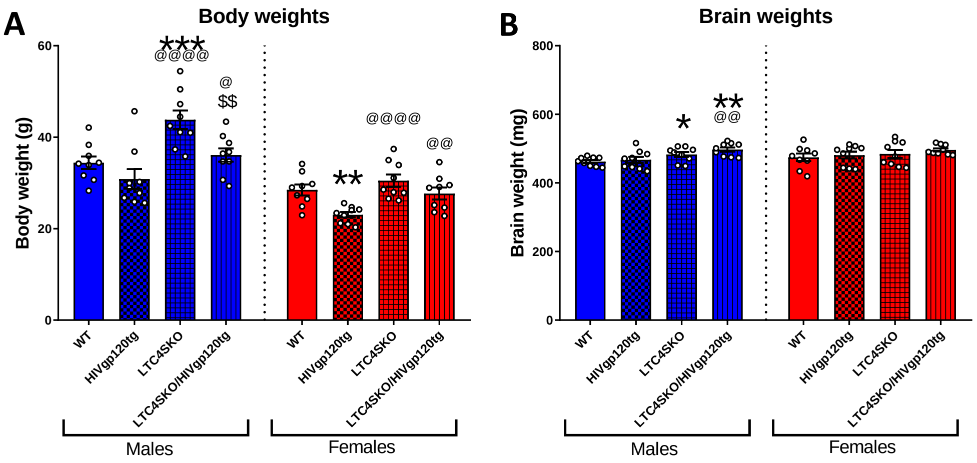

Figure S2. Brain and body weight alterations resulting from HIVgp120tg and LTC4SKO at 12 months of age. Body weights mass in grams of male and female mice at 12 months of age (**A**) and respective brain weight in milligrams (**B**). Male (blue) and female (red) data sets shown separated by a dashed black line. Values in graphs are mean ± s.e.m.; n = 9 per genotype, 12 months of age. Individual data points are represented in the graphs by an ◯ symbol. ANOVA followed by Fisher's PLSD post hoc test for males and females were analyzed independently and graphs are shown on the same axis to illustrate gender-related differences. P-value level of significance is as follows: * $p \leq 0.05$, ** $p \leq 0.01$, *** $p \leq 0.001$, @ $p \leq 0.05$, @@ $p \leq 0.01$, @@@@ $p \leq 0.0001$, $$ $p \leq 0.01$; while P-value significance correlations are symbolized as follows: * indicates significances from the WT, @ indicates significances from HIVgp120tg, and $ indicates significances from LTC4SKO mice.

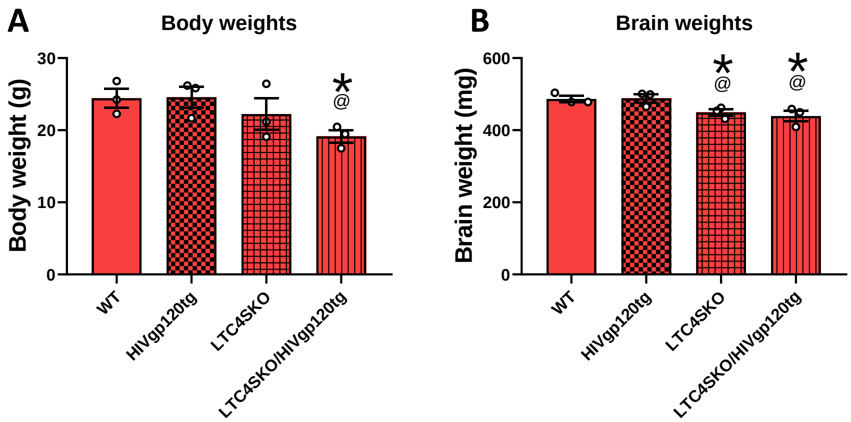

Figure S3. Brain and body weight alterations resulting from HIVgp120tg and LTC4SKO among females harvested for shotgun lipidomics at 6 months of age. Body weights mass in grams of female mice (**A**) and respective brain weight in milligrams (**B**). Values in graphs are mean ± s.e.m.; n = 3 per genotype, 6 months of age. Individual data points are represented in the graphs by an ◯ symbol. ANOVA followed by Fisher's PLSD post hoc test for males and females were analyzed independently. The *p*-value level of significance is as follows: * $p \leq 0.05$, @ $p \leq 0.05$; while *p*-value significance correlations are symbolized as follows: * indicates significances from the WT, @ indicates significances from HIVgp120tg.

Table S1. The mass transitions and collision energies used in LCMS/MS data analysis.

Analyte	m/z Transition (precursor/product ions)	Collision Energy (volts)
PGD2	351.2/233.1	-15
PGE2	351.2/271.2	-25
TXB2	369.2 / 169.1	-25
12-HHT	279.2 / 163.1	-25
PGF2α	353.2 / 193.1	-35
6-keto PGF1α	369.2 / 163.1	-35
12-HETE	319.2 / 179.1	-20
15-HETE	319.2 / 219.1	-20
20-HETE	319.2 / 245.1	-20
5-HETE	319.2 / 115.1	-20
LTC4<br>LTC4-ds	624.5 / 272.2<br>629.4 / 272.2	-35<br>-35
LTD4<br>LTD4-ds	495.3 / 177.1<br>500.4 / 177.1	-35<br>-35
LTE4<br>LTE4-ds	438.3 / 333.2<br>443.3 / 338.2	-35<br>-35
AA	438.3 / 333.2	-35
